# Cardioembolism and Involvement of the Insular Cortex in Patients with Ischemic Stroke

**DOI:** 10.1371/journal.pone.0139540

**Published:** 2015-10-21

**Authors:** Jihoon Kang, Jeong-Ho Hong, Min Uk Jang, Beom Joon Kim, Hee-Joon Bae, Moon-Ku Han

**Affiliations:** 1 Department of Neurology, Samsung Changwon Hospital, Sungkyunkwan University School of Medicine, Changwon, Korea; 2 Department of Neurology, Dongsan Medical Center, Keimyung University School of Medicine, Daegu, Korea; 3 Department of Neurology, Chuncheon Sacred Heart Hospital, Hallym University School of Medicine, Chuncheon, Korea; 4 Department Neurology, Cerebrovascular Center, Seoul National University Bundang Hospital, Seoul National University, Seongnam, Korea; Stanford University, UNITED STATES

## Abstract

**Background:**

To evaluate whether topographical characteristics of insular involvement in ischemic stroke are associated with cardioembolism.

**Methods and Findings:**

A consecutive series of patients hospitalized for ischemic stroke within 7 days of symptom onset were identified. Based on diffusion-weighted imaging, we included those who had ischemic lesions in the middle cerebral artery (MCA) territory. Each patient was assigned to one of two groups based on the presence or absence of insular involvement. The primary outcome was the frequency of cardioembolism, which was compared based on insular involvement. Of 1,311 patients with ischemic stroke in the MCA territory, 112 had insular involvement (8.5%). The frequency of cardioembolism in patients with insular involvement (52.7%) was significantly higher than that in patients without insular involvement (30.4%, *P* < 0.001). Although insular involvement was associated with a severe baseline National Institutes of Health Stroke Scale score (13 vs. 4), it did not independently affect the 3-month functional outcome.

**Conclusions:**

In cases of stroke in the MCA territory, involvement of the insular cortex may be associated with a risk of cardioembolism.

## Introduction

The insula is located at the invaginated portion of the frontal cortex and is involved in various higher cortical functions, such as motor control, homeostasis, and interceptive awareness [1]. Damage to the insular cortex can result in several types of neurogenic signs and symptoms, including various systemic somatosensory symptoms, a swallowing disorder, and cerebrogenic arrhythmia [2, 3].

After stroke, patients with insular involvement demonstrate a higher risk of mortality and unfavorable outcomes compared to those without insular involvement [4, 5]. At the acute stage of ischemic stroke, insular involvement may indicate a higher risk of conversion of salvageable penumbra to irreversibly damaged tissue [6].

Insular involvement might be highly associated with the specific pathomechanism of ischemic stroke [7, 8]. The insula is supplied directly by the proximal portions of the two main branches of the middle cerebral artery (MCA; M2), where they abruptly arise from the main stem (M1) at a right angle. This topographical characteristic of the insula could enable an embolism, especially a cardioembolism (CE), to occlude in the transition region between the M1 and M2 [9, 10]. Although this hypothesis may be useful for the early diagnosis and development of a therapeutic plan, it has rarely been examined.

In the present study, we aimed to determine whether CE may be the major cause of insular involvement in ischemic stroke in the MCA territory.

## Methods

### Approval of the standard protocol and patient consent

This study was approved by the local institutional review board (IRB) of Seoul National University Bundang Hospital, Republic of Korea. The need for written informed consent was waived owing to the retrospective and observational design of the study, which posed no potential harm to the enrolled patients. Patient records and information were anonymized and de-identified prior to analysis.

### Subjects

A consecutive series of 2,770 patients who were hospitalized between December 2004 and July 2011 because of ischemic stroke within 7 days of symptom onset were identified in the institutional stroke registry [11]. Of these, 1,715 patients whose ischemic lesions were located in the MCA territory were selected by reviewing the stroke registry and formal imaging reports written by institutional neuroradiologists. We excluded patients whose stroke mechanism had been determined as small vessel occlusion and who had not been examined with diffusion-weighted imaging (DWI) and magnetic resonance angiography (MRA).

### Data collection and outcome definitions

Insular involvement in the study cases was identified in two steps. First, formal imaging reports were collected from the institutional electronic medical records (EMR) and searched for the terms “insula,” “insular cortex,” “subinsula,” and “extreme capsule” with an automatic keyword searching software integrated in the EMR system.

Second, neurologists (J.K., H.H., and M.K.H.) independently reviewed brain MRIs of subjects selected in the first step and made the final assessments of insular involvement based on cerebral hemisphere templates [12]. We used DWI for ascertaining the insular involvement and MRA for evaluating the stenosis or occlusion status of the cerebral artery.

**Fig 1:**
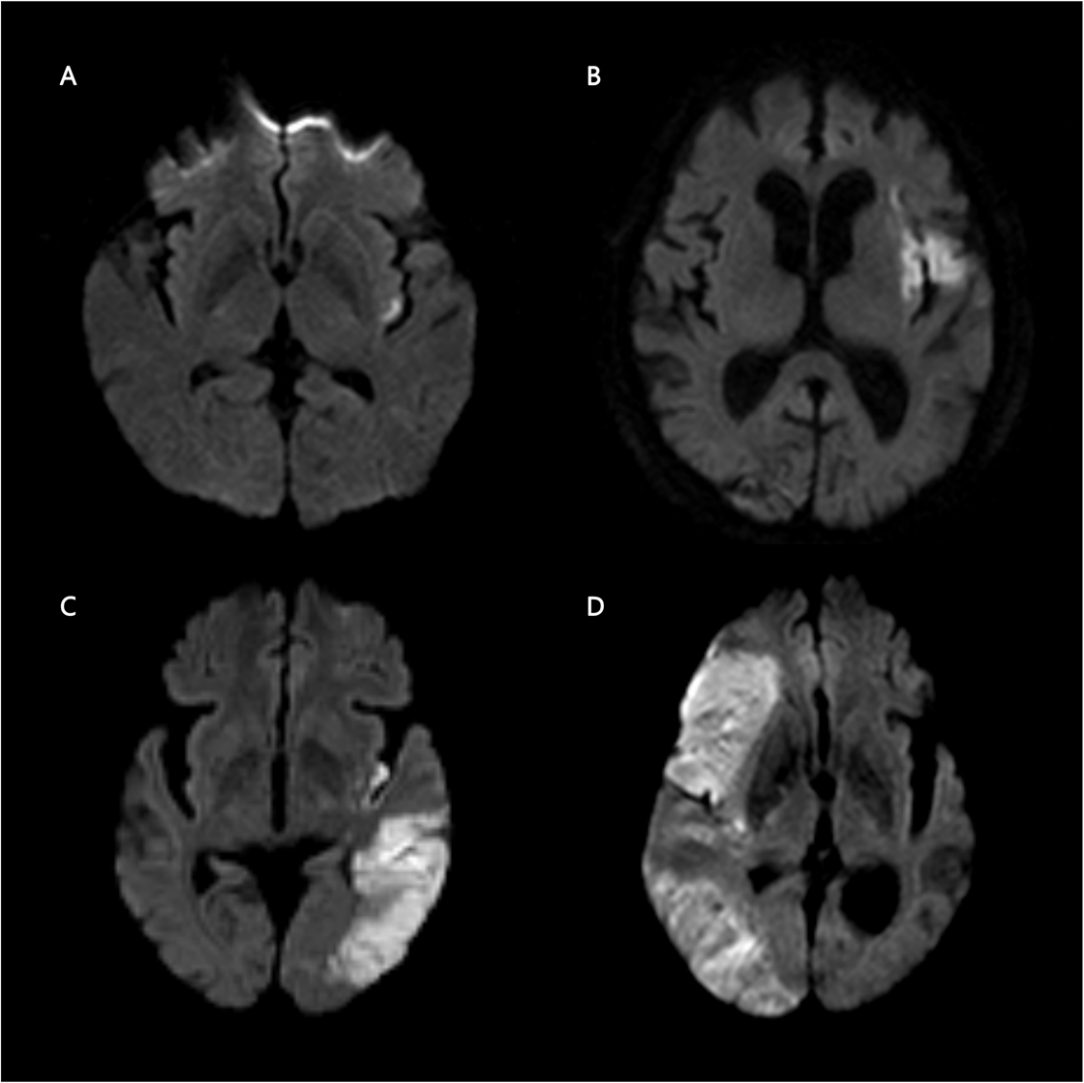
Patterns of insular involvement in ischemic stroke. High signal intensity on diffusion-weighted images illustrates the patterns of insular involvement: isolated insula (A), insula plus adjacent region (B), insula plus remote region (C), and large territorial infarction (D).

Patients with insular involvement were classified into 4 groups according to its pattern: isolated insula (IS), insula plus adjacent region (IA), insula plus remote region (IR), or large territorial infarction (more than one-third of the MCA territory; IL) (Fig 1). The adjacent region included the operculum, lenticulostriate nucleus, extreme capsule, and claustrum, whereas the remote region involved other areas within the MCA territory except for those assigned to the adjacent region.

With regard to the presence and location of symptomatic stenosis or occlusion of the cerebral artery based on MRA results, the patients were categorized as follows: no vascular lesion, proximal vascular lesion, or distal vascular lesion. A proximal vascular lesion was defined as a symptomatic stenosis or occlusion located in the region from the proximal internal carotid artery to the proximal half of the main stem of the MCA, and these arteries were considered a possible embolic source. A distal vascular lesion was defined as a symptomatic stenosis or occlusion located in the region from the distal half of the M1 to the proximal portion of the M2, and these arteries were considered to be associated with the risk of in situ thrombosis.

Demographic, medical, and stroke data including age, baseline National Institutes of Health Stroke Scale (NIHSS) score, stroke mechanism, and presence of diabetes, hypertension, dyslipidemia, and atrial fibrillation were extracted from the stroke registry. The primary outcome was the stroke mechanism, which was assigned by the neurologists based on the Trial of Org 10,172 in Acute Stroke Treatment (TOAST) classification [13]. CE was assigned when the ischemic lesions were from an embolism of cardiac origin in the absence of other embolic conditions and significant stenosis of the relevant cerebral artery on various angiographic studies.

Upon approval from the IRB, we also collected the 3-month modified Rankin scores (mRS) as the functional outcome. As a part of an institutional quality-of-care monitoring program for hospitalized stroke patients, the data on the 3-month functional status were prospectively recorded via direct observation, review of medical records, and telephone interviews. We defined the secondary outcome as a favorable functional status (mRS, 0–2).

### Statistical analysis

Collected data are reported as number (percentage), mean ± standard deviation, or median (interquartile range, IQR) where appropriate. Baseline characteristics were compared between patients with and without insular involvement using a Pearson χ^2^ test for parametric variables, a Whitney-Mann U-test for non-parametric variables, and a *t*-test for continuous variables. Comparison of primary and secondary outcomes with regard to insular involvement was performed using a Pearson χ^2^ test. For the secondary outcome, we estimated the adjusted odds ratio of insular involvement for a favorable functional status at 3 months. Based on potential variables that could affect the functional outcome that we identified from previous publications [13–15], we identified certain imbalanced variables with insular involvement in bivariate analysis (*P* < 0.2) and adjusted for these as confounders (*P* < 0.2). All probability values are 2-sided and considered statistically significant at <0.05. All analyses were performed using SPSS version 18.0 (SPSS Inc., Chicago, IL).

## Results

A total of 1,311 patients with MCA territorial infarction were enrolled. The mean age was 67.7 ± 13.4 years, and men comprised 58.4% of the patients. The median baseline NIHSS score was 5 (IQR, 2–11), and 270 patients (20.6%) underwent some type of recanalization treatment at the hyperacute stage. Stroke mechanisms were classified as follows: large artery atherosclerosis (LAA, 41.6%), CE (32.3%), other determined etiology (3.5%), and undetermined (UD, 22.6%).

Insular involvement was detected by DWI in 112 patients (8.5%). With regard to the pattern of insular involvement, IR was most commonly observed (32.7%), followed by IA (32.0%), IL (26.2%), and IS (4.1%) (Fig 1). Overall, patients with insular involvement demonstrated higher baseline stroke severity and atrial fibrillation rates, whereas some well-known atherosclerosis risk factors, such as hypertension, diabetes, and dyslipidemia, were not significantly different compared with patients without insular involvement (Table 1).

**Table 1:** Comparison of baseline characteristics of patients with and without insular involvement.

Variables	Without insular involvement (n = 1,199)	With insular involvement (n = 112)	*P* value^a^
Male sex	699 (58.3%)	66 (58.9%)	0.90
Age, years	67.5 ± 13.4	70.0 ± 13.1	0.06
Baseline NIHSS score	4 (2–10)	13 (7–18.8)	<0.001
Hypertension	723 (60.3%)	67 (60.4%)	0.99
Diabetes mellitus	319 (26.6%)	24 (21.4%)	0.23
Dyslipidemia	189 (15.8%)	16 (14.3%)	0.68
Smoking	485 (40.5%)	41 (38.6%)	0.43
Arial fibrillation	167 (13.9%)	29 (25.9%)	0.001
Reperfusion treatment^b^	224 (18.7%)	46 (41.1%)	<0.001

Data are presented as number of patients (percentage), mean ± SD, or median (interquartile range).

^a^ *P* values were calculated using Pearson χ^2^ test, Whitney Mann U test, or *t*-test (see Methods).

^b^ Reperfusion treatment consisted of intravenous thrombolysis, intra-arterial treatment, and combined treatment.

The distribution of stroke mechanisms was statistically different with regard to insular involvement (*P* < 0.001). In particular, the frequency of CE in patients with insular involvement (52.7%) was higher than that in patients without insular involvement (30.4%) (Table 2). Depending on the pattern of insular involvement, the frequencies of CE ranged from 44.8% to 80.0%, but the difference between the patterns was not statistically significant (P = 0.18, Table 3).

**Table 2:** Comparison of stroke mechanisms with regard to insular involvement.

Stroke mechanism	Without insular involvement (n = 1,199)	With insular involvement (n = 112)
Large artery disease	510 (42.5%)	36 (32.1%)
Cardioembolism	364 (30.4%)	59 (52.7%)
Other determined	42 (3.5%)	4 (3.6%)
Undetermined		
Two or more	50 (4.2%)	2 (1.8%)
Negative	201 (16.8%)	8 (7.1%)
Incomplete	32 (2.7%)	3 (2.7%)

Data are presented as number of patients (percentage).

**Table 3:** Distribution of stroke mechanisms with regard to patterns of insular involvement.

Stroke mechanism^a^	IS (n = 5)	IA (n = 35)	IR (n = 43)	IL (n = 29)
Large artery disease	1 (20.0%)	8 (22.9%)	18 (41.9%)	9 (31.0%)
Cardioembolism	4 (80.0%)	21 (60.0%)	21 (48.3%)	13 (44.8%)
Other determined	0 (0.0%)	0 (0.0%)	2 (4.7%)	2 (6.9%)
Undetermined	0 (0.0%)	6 (17.1%)	2 (4.7%)	5 (17.2%)
2 or more	0 (0.0%)	1 (2.9%)	0 (0.0%)	1 (3.4%)
Negative	0 (0.0%)	5 (14.3%)	2 (4.7%)	1 (0.5%)
Incomplete	0 (0.0%)	0 (0.0%)	0 (0.0%)	3 (10.3%)

Data are presented as number of patients (percentage). IS, insular isolated; IA, insular plus adjacent region; IR, insular plus remote region; IL, insular plus large territorial region

^a^Stroke mechanism was determined based on the Trial of Org 10,172 in Acute Stroke Treatment (TOAST) classification [13].

A post-hoc analysis was used to investigate the location of symptomatic steno-occlusive lesions in patients who had insular involvement and were assigned to the LAA group of stroke mechanisms (n = 36). Of these patients, 75% had proximal vascular lesions located in the region from the proximal internal carotid artery to the proximal half of the M1, whereas the remaining 25% had vascular lesions between the distal half of the M1 and proximal part of the M2.

In the bivariate analysis, patients with insular involvement had significantly poorer functional status at 3 months compared with those without insular involvement (61.3% versus 37.4%, *P* < 0.001). However, after adjustment for age, baseline NIHSS score, atrial fibrillation, and recanalization treatment, insular involvement was not associated with the 3-month functional outcome (adjusted odds ratio, 0.79; 95% confidence interval, 0.47–1.32).

## Discussion

The results suggest that CE is the primary stroke mechanism in patients with insular involvement (approximately 50% of cases), while LAA constitutes the most common cause of ischemic stroke in the MCA territory. In addition, the data also indicate that arterial embolism may be responsible for insular involvement in most patients with LAA.

A previous study of 150 subjects with MCA territory ischemic stroke conducted in the United States described the clinical features of patients with insular involvement and proposed a greater association between insular involvement and CE (55.6% with insular lesions vs. 37.2% without insular lesions) [7]. This higher frequency of CE in patients with insular involvement was somewhat surprising, given that a Korean epidemiologic study found that CE explained only 11% of total ischemic stroke cases [16]. Of note, a more recent statistical report demonstrated a greater frequency of CE in Korean stroke subjects (20%) [17], suggesting a stronger association between insular involvement and this stroke mechanism.

CE was consistently the most common cause of ischemic stroke for all insular lesion patterns. In particular, CE was detected in 80% and 60% of patients with IS and IA patterns, respectively, which supports the study hypothesis because the IS and IA patterns are more likely to result from occlusion of the transitional segment from the distal part of M1 to the proximal part of M2 by an embolus from the heart.

With regard to clinical practice, the association between CE and insular involvement may be useful for developing diagnostic approaches and deciding treatment strategies at the acute stage of ischemic stroke. Rapid detection of CE through the use of intensive studies, such as transthoracic echocardiography, transesophageal echocardiography, and multi-direction computed tomography, is important for determining the stroke etiology [18], and the results of the present study might help to select patients who would benefit from these extensive examinations. Moreover, our data is potentially useful for planning secondary prevention therapy, which can reduce the high stroke recurrence rate within the first month [19]. Finally, an association might exist with arterial embolism in patients with symptomatic steno-occlusive vascular lesions. Accordingly, intensive monitoring with transcranial sonography [20, 21] could be used to detect micro-emboli, and dual antiplatelet therapy could be utilized [22].

In the present study, patients with insular involvement showed high baseline neurologic impairment compared with the patients without insular involvement (NIHSS score, 13 vs. 4), which is consistent with a previous report (NIHSS score, 13.5 vs. 6) [7]. However, in contrast to other published data, an independent association between insular involvement and the 3-month functional outcome did not exist [4, 5]. This finding suggests that insular involvement is related to severe neurologic deficits but does not additionally influence the stroke outcome.

This study has several limitations, primarily related to the study design. First, it was an observational study with a retrospective design, which could increase the risk of selection bias. However, the risk was likely reduced because our study subjects were selected from a prospective stroke registry. Second, there was a considerable proportion of patients with the UD stroke mechanism, which could affect the overall distribution. Our hospital uses a unified classification algorithm to determine the mechanism of ischemic stroke, resulting in an unintentional imbalance between the proportion of patients with each mechanism [23]. Third, insular involvement could provoke cerebrogenic arrhythmia, which may have affected the frequency of CE [2, 7]. Because exact values of premorbid CE risk are not available, some uncertainty regarding the causality will remain. However, the association between insular involvement and CE was demonstrated clinically, which might be useful in practice.

In conclusion, the results of this study suggest a high association between cardioembolic stroke and insular involvement in MCA territory ischemic stroke. This finding provides clinical insights into stroke etiology and might be helpful for establishing a diagnosis.

## Supporting Information

S1 Dataset Database containing patient information. (SAV)

S1 Table Summary of variables in the dataset. (DOCX)
