# Supplementary material for: Cardioembolism and Involvement of the Insular Cortex in Patients with Ischemic Stroke
Source: PLoS One. 2015 Oct 21;10(10):e0139540. doi: 10.1371/journal.pone.0139540 (PMC4619548; doi:10.1371/journal.pone.0139540)
Supplement: S1 Table — (DOCX) [file pone.0139540.s002.docx]

**Supporting information**

We uploaded the database as the supporting information. Patients were de-identified and removed any information to be specified a patient. The variables were summarized (S1_Table. Summary of variables in the database).

S1_Table. Summary of variables in the database

| Variables | Labels | Coding |
| --- | --- | --- |
| ins_ves | Location of vascular lesion | 0: no vascular lesion  1: vascular lesion at ICA or proximal M1  2: vascular lesion beyond distal M1 |
| insula | Insular involvement | 0: without insular involvement 1: with insular involvement |
| ins_class | Pattern of insular involvement | 1: Insular isolated  2: Insular with adjacent region  3: insular with remote region  4: insular with large territory |
| fx_outcome | Group of 3 month functional outcome | 0: poor outcome  1: good outcome |
| mrs3_1 | 3month functional outcome (mRS) | 1. No symptom at all 2. No significant disability despite symptoms 3. Slightly disability: unable to carry out all previous activities but able to look after own affairs without Assistance 4. Moderate disability: requiring some help, but able to walk without assistance 5. Moderate to severe disability: unable to walk without assistance, and unable to attend to own bodily needs without assistance 6. Severe disability: bedridden, incontinent, and requiring constant nursing care and attention 7. Death |
| thromb1 | Thrombolysis | 0: no thrombolysis, 1: thrombolysis |
| hx_stroke | Prior stroke history | 0: No, 1: Yes |
| l_ica | Ischemic lesion at ICA territory | 0: No, 1: Yes |
| l_mca | Ischemic lesion at MCA territory | 0: No 1: Yes |
| toast | Stroke mechanism classification, TOAST | 1: Large artery disease  2: Small vessel occlusion  3: Cardioembolism  4: Other determined  5: Undetermined |
| male | Sex | 0: Female, 1: Male |
| age | Age | Continuous |
| nih_initial | Baseline NIH stroke scale score | Continuous |
| htn | Hypertension | 0: No, 1: Yes |
| dm | Diabetes mellitus | 0: No, 1: Yes |
| hl | Dyslipidemia | 0: No, 1: Yes |
| af | Atrial fibrillation | 0: No, 1: Yes |
| smok | Smoking | 0: No, 1: Yes |
| ins_size | Size of insular lesion | 1: less than half of insular cortex  2: more than half of insular cortex |
| a_mca_1 | Vascular lesion at MCA | 0: No, 1: Yes |
| a_eica_1 | Vascular lesion at extracranial ICA | 0: No, 1: Yes |
| a_iica_1 | Vascular lesion at intracranial ICA | 0: No, 1: Yes |
